# Commentary: Assessment of Hypertension Using Clinical Electrocardiogram Features: A First-Ever Review

**DOI:** 10.3389/fmed.2021.691330

**Published:** 2021-07-05

**Authors:** Tatsuya Sato

**Affiliations:** ^1^Department of Cellular Physiology and Signal Transduction, Sapporo Medical University School of Medicine, Sapporo, Japan; ^2^Department of Cardiovascular, Renal, and Metabolic Medicine, Sapporo Medical University School of Medicine, Sapporo, Japan

**Keywords:** electrocardiogram, blood pressure monitoring, QT dispersion, Tpeak–tend, ventricular repolarization heterogeneity, wearable device

In their recently published systematic review entitled “Assessment of Hypertension Using Clinical Electrocardiogram Features: A First-Ever Review” Bird et al. (1) addressed and discussed whether indices of an electrocardiogram (ECG) alone can estimate not only individuals with hypertension but also their blood pressure values. Although recent technological advances might enable easy evaluation and monitoring of biological information including cardiovascular dynamics using smartwatches and wearable devices, caution is needed for attempting to evaluate blood pressure values using only conventional indices of an ECG.

QTc dispersion and Tpeak-Tend interval, which the review revealed had strong associations with hypertension, are considered as indicators that reflect ventricular repolarization heterogeneity (VRH) (2, 3). In fact, Yee et al. (4) demonstrated that an acute increase in cardiac afterload induced by administration of phenylephrine enhanced QTc dispersion independently of changes in heart rate. Also, it was shown that treatment with some anti-hypertensive agents reduced the enhanced QT/QTc dispersion in patients with hypertension (5, 6), suggesting that changes in blood pressure values are associated with indices of VRH. However, one major problem in trying to estimate blood pressure values from indices of VRH is that increased VRH is not a specific feature for hypertension or elevated blood pressure. Malik et al. (7) demonstrated that QTc dispersion is increased in individuals with cardiomyopathy, ischemic heart disease, long QT syndrome, and left ventricular hypertrophy of various origins. Metabolic diseases including diabetes without hypertension (8), obesity (9), and even the presence of insulin resistance without diabetes or hypertension (10) have been reported to be associated with increased indices of VRH. Hypokalemia (11), one of the most common serum electrolyte imbalances, is also a well-known factor leading to the enhancement of VRH. Thus, since increased VRH is associated with not only heart disease but also common comorbidities, it is difficult to estimate blood pressure level or the presence of hypertensive heart disease based on the degree of VRH alone unless a way for differentiating condition-specific changes in VRH is established.

In addition to VRH, it was stated in the review that P wave dispersion (12), which is defined by the difference between the maximum and minimum P wave intervals in a standard 12-lead ECG, is a promising index for estimating elevated blood pressure. However, as in the case of VRH, P wave dispersion is not specific for hypertension or elevated blood pressure but is greatly affected by left atrial morphology or enlargement for various reasons. Besides, a recent study by Zawadzki et al. (13) indicated that P wave dispersion measured by ECG recording with faster paper-speed and increased sensitivity was significantly reduced compared to that measured by standard ECG recording, suggesting that P wave dispersion is just a value due to measurement error and that clinical usefulness for detecting morphological and functional abnormalities of the left atrium can be replaced by P wave interval.

From the viewpoint of easily estimating blood pressure values using wearable devices that are limited to a small number of ECG leads, it remains unclear whether indices of VRH evaluated by wearable devices reflect those measured by a standard 12-lead ECG. Instead, a method for assessing blood pressure values using wearable devices has already been developed by combining ECG waveform and photoplethysmography (14) as the authors mentioned in their review, and the validity of a device using this method for blood pressure measurement has recently been examined (15). Smartwatch-type wrist-cuff oscillometric blood pressure monitoring devices have also become available, although it should be noted that the accuracy of wrist blood pressure measurement is affected by the wrist position (16) and by exercise-induced increase in autonomic nerve activity (17). Thus, methods for non-invasive blood pressure monitoring using wearable devices are being investigated and established. Instead, methods for detecting the onset of cardiovascular disease or events by using real-time ECG recording by wearable devices are expected in clinical settings. For example, detection of the onset of atrial fibrillation with a smartwatch has already been investigated (18) and its validity has been discussed (19). In addition, power spectral analysis of heart rate variability evaluated by wearable devices, which can estimate the balance in autonomic nerve activity, may be able to reflect the severity of cardiovascular disease including heart failure (20). Furthermore, values of Tpeak-Tend and Tpeak-Tend/QT, which are indicators of VRH that can be evaluated with only single-lead ECG, may predict the future onset of malignant arrhythmia (21). Since it has recently been reported that an ECG evaluated by artificial intelligence can contribute to the evaluation and management of specific cardiovascular diseases and conditions (22), application of artificial intelligence may be more promising for estimating hypertension than using conventional indices of an ECG. It should be noted that the issue of noise and artifacts in long-term monitoring by wearable devices may affect the accuracy of any prediction method. Further research is needed to establish clinically appreciable ways of using an ECG recorded by a wearable device to predict cardiovascular disease or events.

Collectively, the review written by Bird et al. raised important issues regarding the possibly novel application of ECG using recent technological advances. It should be noted that there are silent confounding factors to be considered when trying to evaluate blood pressure values using conventional indices of VRH alone. Classical tools for detecting underlying cardiovascular conditions should be complemented by the use of emerging technologies.

## Author Contributions

The author confirms being the sole contributor of this work and has approved it for publication.

## Conflict of Interest

The author declares that the research was conducted in the absence of any commercial or financial relationships that could be construed as a potential conflict of interest.
